# Overlooked and underestimated: the prevalence and impact of mechanical cement-related complications in unicompartmental knee arthroplasty

**DOI:** 10.1186/s42836-026-00439-x

**Published:** 2026-10-14

**Authors:** Zhenggui Yu, Baijian Wu, Yiming Lin, Ruan Chen, Guochang Bai, Xinyu Fang, Wenming Zhang, Zida Huang

**Affiliations:** 1 Department of Orthopaedic Surgery, the First Affiliated Hospital, Fujian Medical University, Fuzhou, 350005 China; 2 Department of Orthopaedic Surgery, National Regional Medical Center, Binhai Campus of the First Affiliated Hospital, Fujian Medical University, Fuzhou, 350212 China

**Keywords:** Unicompartmental knee arthroplasty, Mechanical cement-related complications, Residual cement extrusion, Free cement fragmentation, Bone cement

## Abstract

**Background:**

This study aims to elucidate the prevalence, clinical manifestations, and potential risk factors of residual bone cement following unicompartmental knee arthroplasty (UKA) to inform effective diagnostic and therapeutic strategies.

**Methods:**

This retrospective analysis included 321 patients from a single centre who underwent UKA. Residual bone cement was diagnosed using postoperative imaging and classified as residual cement extrusion (RCE) or free cement fragmentation (FCF). Patient data, including clinical records, laboratory findings, and selected management strategies, were collected to analyse risk factors for residual bone cement. The primary outcomes were the prevalence and clinical features of residual bone cement; secondary outcomes included risk factor identification and management outcomes.

**Results:**

Residual bone cement occurred in 84 patients (26.16%), 66 (20.56%) of whom had RCE, and 18 patients (5.61%) had FCF. All RCE patients were asymptomatic or experienced only mild discomfort (66 in 66, 100%). Six patients with FCF required additional surgical intervention due to pain and/or impaired mobility. The thinner polyethylene insert emerged as a risk factor for postoperative residual bone cement (OR 2.59, 95% CI 1.38–4.98, *p* = 0.005).

**Conclusions:**

The prevalence of residual bone cement following UKA is notably higher than previously recognized. Residual bone cement occurred in 84 patients (26.16%) in our study. As CT was performed only in symptomatic patients, this figure likely represents a lower-bound estimate of the true prevalence. Although most patients with residual cement are asymptomatic, further surgery is needed when FCF causes consistent pain and affects joint function. Arthroscopic removal appears effective for fragments in symptomatic patients. A thinner insert is a risk factor for residual bone cement.

## Introduction

Unicompartmental knee arthroplasty (UKA) has become increasingly popular as a viable option for the treatment of unicompartmental knee osteoarthritis (OA) [1]. UKA is a suitable alternative to total knee arthroplasty (TKA). Compared with TKA, UKA involves a smaller incision and preserves more musculoskeletal tissue, providing a basis for rapid postoperative recovery. Previous studies revealed good clinical outcomes with UKA [2–5]. Chen et al. reported that patients who underwent Oxford UKA had significantly better postoperative numerical rating scale (NRS) scores and knee Society scores (KSS) [2].

Complications after UKA have been reported. According to the French Hip and Knee Society (Société française de la hanche et du genou) [SFHG], the most common causes of UKA failure included loosening (45%), osteoarthritis progression (15%), and wear (12%). Though some adverse events are less frequently reported, they can impair joint function and quality of life. Among these, free cement fragments have been identified as a neglected complication [6]. These fragments act as intra-articular loose bodies, potentially causing pain, reduced range of motion, and accelerated polyethylene wear, which may ultimately lead to severe mechanical failure [6–10]. However, there are few reports on mechanical complications caused by bone cement after UKA, and most cases are presented in the form of case reports [11–15]. The risk factors for residual bone cement have not yet been established, and the preventive strategies against residual bone cement remain inadequate, thereby hindering surgeons’ understanding of this series of syndromes. Therefore, we report the phenomenon of residual bone cement and postoperative discomfort due to bone cement in UKA. In this study, Residual bone cement following UKA was categorized into two types: residual cement extrusion (RCE) and free cement fragmentation (FCF). RCE was defined as a radiopaque density extrusion shadow around the prosthesis and beyond the component or cortex on imaging. FCF was characterized by its presence in the joint cavity following the removal of cement from the original platform. We define mechanical cement-related complications (MCRC) as cases in which free cement fragmentation causes pain, poor mobility, or even joint locking after UKA. Other severe mechanical complications, such as polyethylene insert dislocation, may also occur in association with this condition.

The primary aim of this study was to review the prevalence and clinical features of residual bone cement after unicompartmental knee arthroplasty (UKA). Secondary aims were to identify potential risk factors, evaluate management outcomes and strategies, and provide clinical guidance for the management of residual bone cement.

## Method

Ethical approval was obtained from the First Affiliated Hospital of Fujian Medical University Ethics Committee. (Approval No: MRCTA, ECFAH of FMU(2019)296). Informed consent was obtained from all participating patients. The study protocol was approved by the local ethics committee and complied with the Declaration of Helsinki. This was a single-centre retrospective study. A total of 321 patients who underwent UKA at a single UKA-qualified medical centre between January 2016 and June 2023 were included in this study. A minimum follow-up of 1 year was available for all patients. The surgical indication was medial unicompartmental osteoarthritis. Experienced surgeons confirmed the surgical indications, which were selected on the basis of local symptoms, signs, serological results, and imaging data. The exclusion criteria were excessive deformity due to OA, inflammatory joint disease, infectious arthropathy, and a history of lower extremity fractures [16].

### Surgical technique

All surgical techniques were performed according to the standard operating procedures by the same medical team who performed more than 30 UKAs per year [16, 17]. The operation was performed under a tourniquet. The tourniquet was typically inflated before skin incision and maintained until complete cement curing. All patients (*n* = 321) received cement from Oxford Mobile unicompartmental prostheses (Zimmer Biomet, Watson, USA). All surgeries were performed by experienced surgeons (≥ 10 years) from the same team, following the same UKA operative protocol, and attempts were made to remove all visible cement. However, despite these efforts, complete clearance—particularly in the posterior compartment around the tibial component—was not always achievable, as reflected in the 26% residual rate observed in this cohort. The bone cement was prepared using a single 40-g pack and was mixed using a closed vacuum mixing system. The cement was applied when it reached the sticky and dough phases. The procedure for prosthetic implantation and bone cement removal is as follows. The Tibial Component: Place a small amount of cement on the tibial bone surface and flatten to produce a thin layer covering the whole undersurface. Insert the component and press down, first posteriorly and then anteriorly, to squeeze out excess cement at the front. Use the right-angled tibial impactor with a small mallet to complete the insertion. Ensure there is no soft tissue under the component. The Femoral Component: Force cement into the large femoral drill hole and fill the concave surface of the femoral component with cement. Apply the loaded component to the condyle and impact with the punch held at 45 degrees to the long axis of the femur. Pressurize the cement by inserting the appropriate feeler gauge with the knee at 45 degrees of flexion and holding the leg in this position. Once the cement has set, remove the feeler gauge. Clear the medial and lateral margins of the component of any extruded cement. The posterior margin was palpated with a curved dissector. After all prostheses have been implanted, the knee joint was irrigated with saline to remove free cement fragments.

The selection of meniscal bearing thickness is primarily based on restoring natural ligament tension and balancing the flexion and extension gaps. First, the flexion gap is measured at 100° of flexion using feeler gauges; the correct thickness is confirmed when the gauge slides in and out smoothly without tilting while maintaining natural ligament tension. Next, the extension gap is measured at 20° of flexion, and the difference between the flexion and extension gaps dictates the amount of femoral bone to be milled in order to equalize them. Finally, with trial components in place, applying a valgus stress at 20° of flexion serves as the ultimate confirmation, where the ideal thickness allows the artificial joint surfaces to distract by 1 to 2 mm.

### Postoperative evaluation and residual bone cement management

**Fig. 1:**
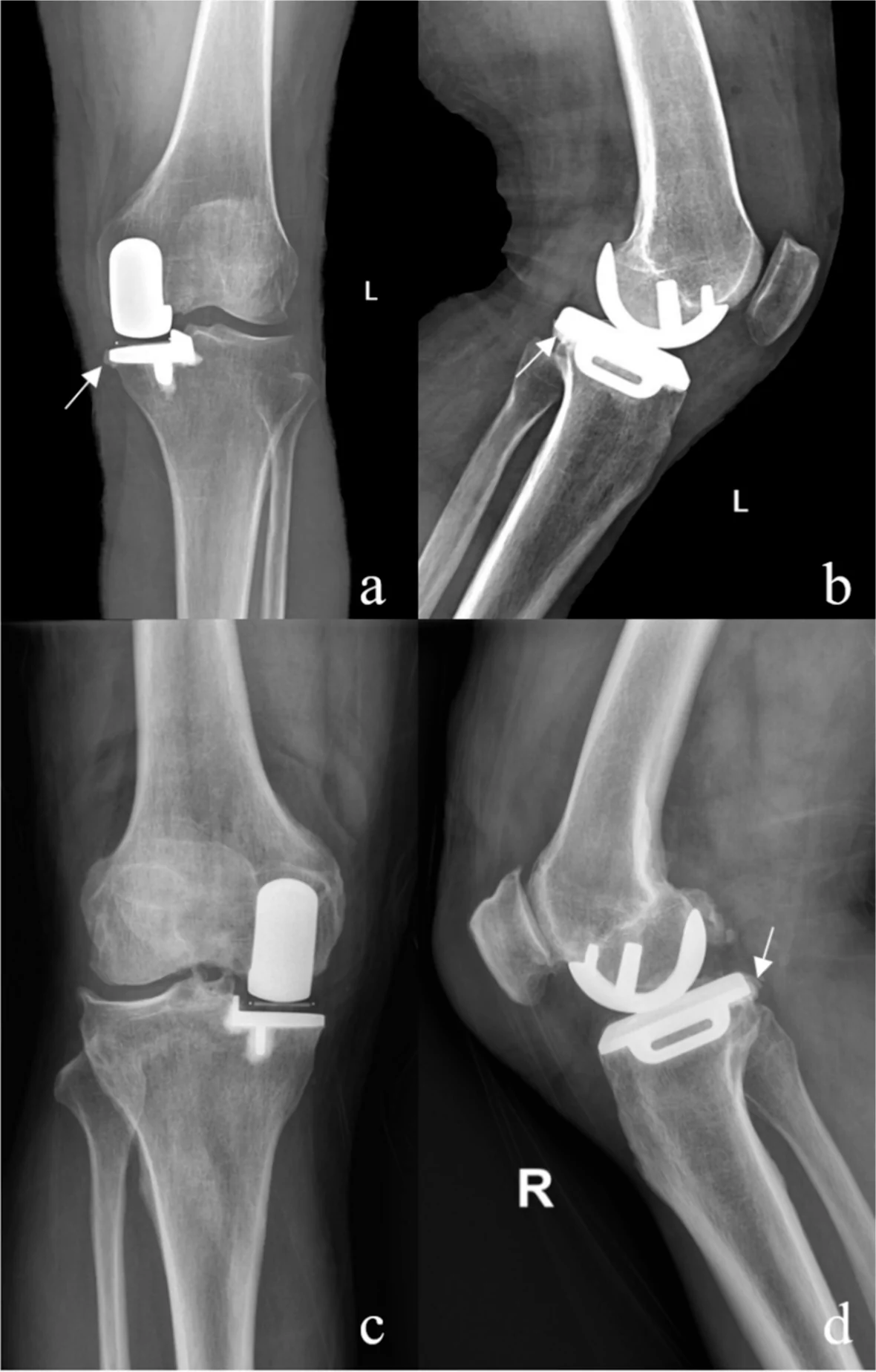
Residual cement after UKA. **a**, **b** Anteroposterior and lateral radiographs showing residual cement extrusion; **c**, **d** Anteroposterior and lateral radiographs of free cement fragmentation

The postoperative management of all patients follows the standard protocol. On the day of surgery, physiotherapy was initiated 6 h postoperatively; Patients were able to ambulate with weight-bearing using a walker on the first postoperative day; Discharge is usually possible within 1–2 days once pain is controlled, vital signs are stable, and the patient can move safely. Serological tests (including Complete blood count, C-reactive protein, and erythrocyte sedimentation rate) and imaging examinations were performed 3 days, 6 weeks, 3 months, and 6 months after the operation and then annually thereafter. These tests are promptly recommended for patients with symptomatic UKA. The radiological diagnosis of residual bone cement was based on postoperative imaging findings and confirmed by one orthopaedic surgeon and one radiologist who reviewed standard AP and lateral view images of the unicompartmental prosthesis. If the two evaluators disagree, another orthopedic surgeon is consulted to assess in order to ensure the objectivity of the results. Residual bone cement was diagnosed using postoperative imaging and classified as residual cement extrusion (RCE) or free cement fragmentation (FCF). RCE was defined as a radiopaque density extrusion shadow around the prosthesis and beyond the component or cortex on imaging (Fig. 1a and b). The assessment of RCE was performed on postoperative anteroposterior and lateral radiographs; we explicitly documented the primary locations of the residual bone cement. FCF was characterized by its presence in the joint cavity following the removal of cement from the original platform, without taking its mobility into consideration (Fig. 1c and d). Cement-related mechanical complications, including dislocation, wear, and subluxation of the prosthesis, were caused by residual bone cement or free bone cement. CT is indicated for symptomatic residual bone cement and its associated signs or symptoms. Symptoms of residual bone cement include pain that persist with necessitate regular analgesic use (VAS ≥ 4), reduced joint mobility (ROM < 90°), or even locking. A foreign body that changes with position may be palpated during physical examination.

The management strategy was stratified based on patients’ symptoms, functional status, and imaging findings. For patients who were asymptomatic or experienced only mild discomfort (defined as VAS < 4 and ROM ≥ 90°) with imaging findings consistent with residual cement extrusion or free cement fragmentation, observation or conservative treatment (including physical therapy and oral analgesics as needed) was recommended as the initial approach. If symptoms persisted or worsened despite adequate conservative therapy, surgical intervention was indicated based on the specific location of the residual material: accessible free fragments within the joint space could be removed arthroscopically, whereas free fragments located in the posterior articular cavity required open surgical removal. Open surgery was performed through the original surgical incision. If the insert shows no wear, the procedure can be concluded after removal of loose cement fragments; if insert wear is present, replacement of the insert is required.

### Outcome measures and categorization method for variables

The primary measures included VAS, KSS, ROM, and the location of residual bone cement, while the secondary measures comprised CRP, ESR, and the management approach. In our surgical practice, we have found that residual bone cement removal is more technically demanding in patients receiving smaller prostheses and inserts. Based on our surgical experience, we categorize the components according to their size. Smaller components were defined as “AA”, “A” and “B” size tibial components, and “Extra small” and “Small” size femoral components. The polyethylene insert of 3- or 4- mm thickness was classified as thin size, while the polyethylene inserts of 5- or 6- mm thickness and those of 7- or 8- mm thickness were classified as middle size and thick size, respectively.

### Statistical analysis

Continuous variables are expressed as means and standard deviations for normally distributed data, and as medians and quartiles for non-normally distributed data. Student’s t-tests or rank-sum tests were used to compare statistical differences in continuous variables. Categorical data are expressed as counts and percentages and were compared with the chi-square test and Fisher’s exact test. Single logistic regression analysis was used to explore the risk factors related to residual bone cement. A *p*-value of less than 0.05 indicated statistical significance. SPSSv26.0 (IBM, Armonk, New York, USA) was used for all analyses.

## Results

A total of 321 patients were included in this study. Residual bone cement was diagnosed in 84 (26.16%) patients, including 66 (20.56%) with RCE and 18 (5.61%) with FCF. The demographic data of the residual bone cement and Normal groups are shown in Table 1, and there were no significant differences between the two groups.

**Table 1:** Demographic data

	**FCF or RCE (*n* = 84)**	**Normal (*n* = 237)**	***p*-value**
Sex, *n* (%)			0.482 ^†^
Male	26(30.95)	62(26.20)	
Female	58(69.05)	175(73.80)	
Side, *n* (%)			0.194 ^†^
Left	40(47.62)	137(57.80)	
Right	44(52.38)	100(42.20)	
Age, y (SD)	66.6(8.09)	65.2(7.84)	0.166 *
BMI, kg/m^2^ (SD)	25.2(3.39)	25.9(3.44)	0.927 *
aCCI (SD)	2.51(1.10)	2.46(1.05)	0.730 *
Mean follow-up time, y (SD)	5.13 (2.47)	5.69 (3.05)	0.133 *

*FCF* free cement fragmentation, *RCE* residual cement extrusion

^†^: Chi-square test

^*^: Independent-samples t-test

The clinical outcomes are detailed in Table 2. Inter-observer agreement was good (Kappa = 0.932, *p* < 0.001). Most cases of RCE or FCF in this cohort were asymptomatic or mildly uncomfortable, and these patients generally had satisfactory outcomes after observation and appropriate conservative treatment. Compared with the early postoperative period, seven patients developed local pain and five experienced limited range of motion compared with the early postoperative period. Of these, 3 patients presented with both pain and decreased mobility, and six patients (those with FCF listed in Table 3) required surgical intervention due to persistent symptoms. 5 patients with FCF underwent arthroscopic removal. A patient with FCF underwent open surgery for the removal of the fragment. Open surgery was performed through the original incision. Intraoperative exploration revealed free cement fragmentation within the posterior articular cavity, with no obvious wear of the prosthesis or the polyethylene insert. Therefore, the procedure was concluded after removal of the residual cement fragments. We conducted long-term follow-up on all patients who underwent surgical removal of free cement fragments. All patients experienced relief of postoperative knee pain and also showed significant improvement in range of motion. The clinical data of the 6 patients who underwent reoperation are shown in Table 3.

**Table 2:** Clinical outcomes

	**FCF or RCE (*n* = 84)**	**Normal (*n* = 237)**	***p*-value**
Mean CRP, mg/L(SD)	4.63 (2.71)	4.55 (3.41)	0.824 *
Mean ESR, mm/h(SD)	25.3 (14.0)	23.9 (12.7)	0.435 *
VAS	1.71 (1.59)	1.51 (1.19)	0.295 *
KSS	79.7 (15.6)	82.4 (9.61)	0.131 *
ROM, °(SD)	117 (18.1)	120 (8.52)	0.085 *
Management, *n* (%)			
Follow-up or conservative management	78 (92.86)	237 (100)	
Arthroscopy or open surgery	6 (7.14)	0 (0)	
Location/Type^†^, *n* (%)			
Posterior to the tibial prosthesis	51 (60.71)	-	
Residual cement in other sites	15 (17.86)	-	
FCF (type)	18 (21.43)	-	

*FCF* free cement fragmentation, *RCE* residual cement extrusion, *CRP* C-reactive protein, *ESR* erythrocyte sedimentation rate, *VAS* visual analogue scale, *KSS* Knee Society Score, *ROM* range of motion

^*^: Independent-samples t test

^†^: Location categories are reported for RCE only; FCF is listed separately by type because fragmentation was not consistently localizable to a single anatomical site

**Table 3:** Details of patients who underwent reoperation

Patient number	Residual bone cement	Sex	Age	Side	Pain	Decreased mobility	Interval (m)	CRP (mg/L)	ESR (mm/h)	Management	Post-op VAS	Post-op ROM, °
1	FCF	Female	68	Left	Yes	No	3	0.6	2	Arthroscopic	1	5–100
2	FCF	Female	74	Right	Yes	Yes	2	1.5	20	Arthroscopic	1	0–105
3	FCF	Female	70	Left	Yes	Yes	48	0.5	2	Open surgery	0	5–110
4	FCF	Female	72	Left	Yes	No	16	4.2	22	Arthroscopic	1	0–100
5	FCF	Male	80	Left	Yes	No	6	1.7	18	Arthroscopic	0	0–110
6	FCF	Female	68	Right	Yes	Yes	9	3.3	31	Arthroscopic	1	5–105

Decreased mobility; range of motion was decreased compared with the early postoperative phase

Interval: time from primary surgery to detection of cement fragments

*FCF* free cement fragmentation

To explore the risk factors for residual bone cement, we compared these 84 patients who were diagnosed with residual bone cement with the remaining 237 normal patients. Logistic regression analysis revealed that the thinner polyethylene insert was a risk factor for residual bone cement (OR 2.59, 95% CI 1.38–4.98, *p* = 0.005). Other factors, such as age, BMI, and the size of the prosthesis implanted in UKA, did not significantly differ. The logistic regression outcomes are detailed in Table 4.

**Table 4:** Possible risk factors for residual bone cement analysed by logistic regression

	**Residual bone cement (*n* = 84)**	**Normal (*n* = 237)**	**OR**	**95% CI**	***p*-value**
Sex, *n* (%)			1.27	0.73 to 2.17	0.398 ^†^
Male	26(30.95)	62(26.20)			
Female	58(69.05)	175(73.80)			
Side, *n* (%)			1.44	0.87 to 2.37	0.155^†^
Left	40(47.62)	137(57.80)			
Right	44(52.38)	100(42.20)			
Age, y (SD)	66.6(8.09)	65.2(7.84)	1.02	0.99 to 1.06	0.158^†^
BMI, kg/m^2^ (SD)	25.2(3.39)	25.9(3.44)	0.94	0.87 to 1.01	0.109^†^
Operation time, min (SD)	110(32.40)	109(29.50)	1.00	0.99 to 1.01	0.773^†^
Tibial prosthesis size, *n* (%)			0.88	0.46 to 1.74	0.699^†^
Small	69(82.10)	199(84.00)			
Large	15(17.90)	38(16.00)			
Femoral prosthesis size, *n* (%)			1.14	0.59 to 2.33	0.701^†^
Small	71(84.50)	196(82.70)			
Large	13(15.50)	41(17.30)			
Polyethylene insert thickness, *n* (%)			2.59	1.38 to 4.98	0.005^†^
Thin	71(84.50)	162(68.40)			
Middle or thick	13(15.50)	75(31.60)			

^†^: Single logistic regression analysis

## Discussion

Minimally invasive arthroplasty reduces soft tissue trauma, preserves the integrity of the quadriceps tendon and patellar tendon, and does not require patellar eversion. Satisfactory prosthesis positioning could effectively improve the efficacy of UKA [18]. In this study, the incidence of residual bone cement was 26.16% (84 of 321); 66 patients exhibited RCE; FCF occurred in 18 cases, which may have originated from incomplete clearance of FCF during surgery or from fragmentation of residual bone cement. Hauptmann et al. reported that the incidence of RCE and FCF was 20.8% (25 in 120) [6]. Owing to the lack of systematic reviews of residual bone cement, early studies suggested that residual cement was a rare complication of UKA [13, 14]. Since residual bone cement does not cause notable clinical manifestations such as pain or limited mobility in the majority of cases, we do not regard RCE as a clinical complication but rather as a clinical phenomenon. Only when the retained cement causes symptoms do we define the condition as MCRC. Recent studies have shown that MCRC is not a rare postoperative complication [6, 16, 19]. It is of great clinical importance to recognize the prevalence of residual bone cement. We believe that the prevention of residual bone cement and timely intervention could indeed improve patient satisfaction after UKA.

**Fig. 2:**
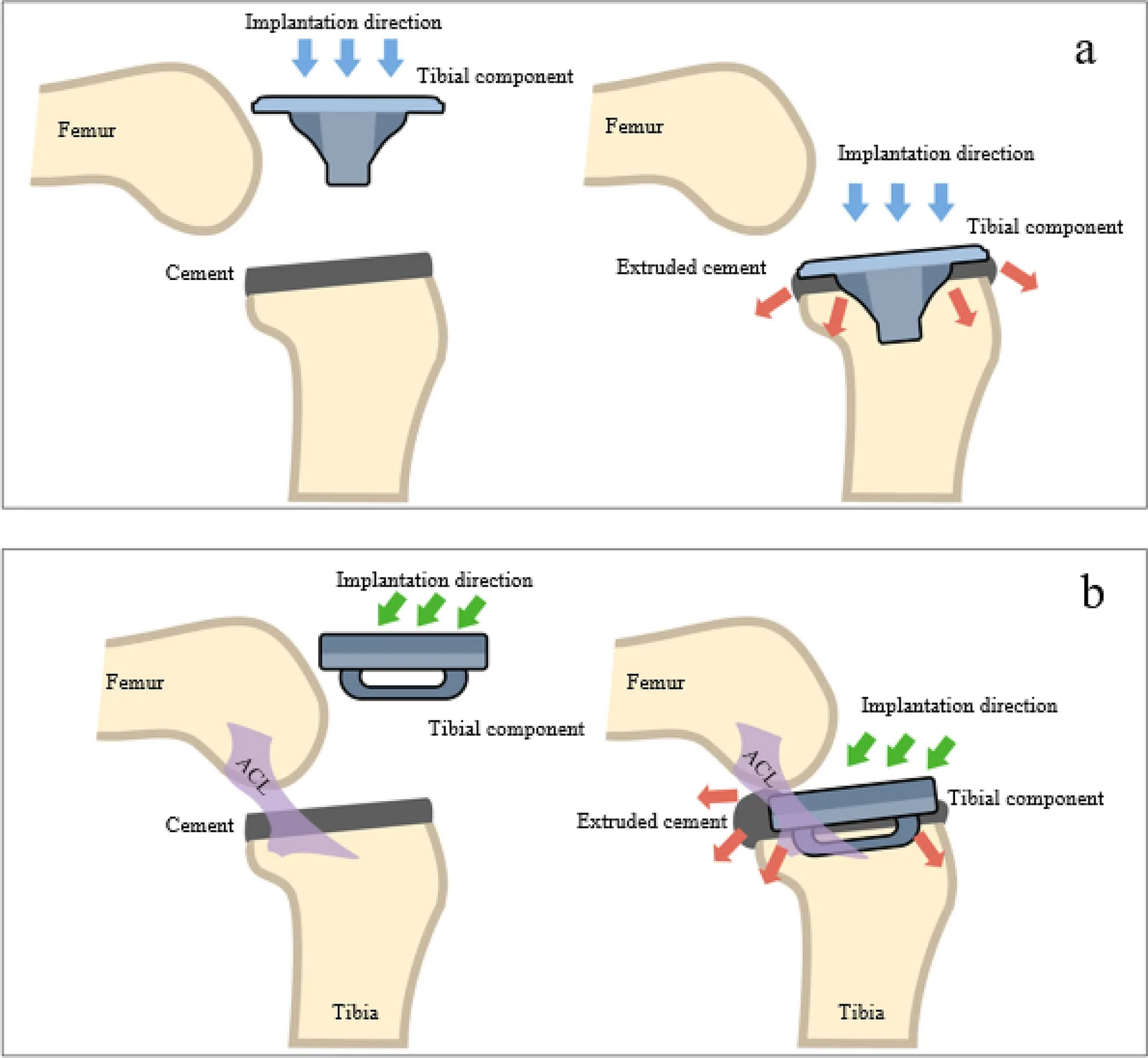
Cement extrusion caused by the insertion of tibial components. **a** Vertical insertion of the tibial component in TKA results in uniform extrusion of the cement in all directions. **b** The anterior–superior insertion of the tibial component in UKA results in more backward extrusion of the cement. ACL, Anterior cruciate ligament

Most studies revealed that residual bone cement is caused by a reduced view of the posterior part of the joint [14, 16, 20]. Although minimally invasive incisions are associated with unnecessary trauma during surgery, the narrow space also increases the difficulty of surgery, particularly implant placement. Similarly, some surgical tools cannot pass through this small area, which explains why residual cement is often found in the posterior part of the joint [16]. The size of the polyethylene insert in our study was a risk factor for postoperative residual bone cement, which supported the previous idea to some extent. A thinner spacer means a narrower space between the femur and tibia. Although proactively adjusting the size of the polyethylene insert is inappropriate, intensified follow-up is warranted in patients receiving a smaller insert. Timely intervention should be performed once clinical symptoms emerge, so as to prevent serious clinical complications. Yang et al. have found that hybrid fixation is associated with a significantly lower probability of residual bone cement compared with cemented fixation [21]. This may be attributable to the fact that hybrid fixation allows sufficient surgical time for removal of residual cement. Hiranaka et al. found that Japanese patients who underwent cementless UKA had a higher rate of tibial plateau fractures [22]. For the Chinese population, whose skeletal morphology is similar to that of the Japanese, the use of cemented or hybrid fixation prostheses remains a reasonable option. Hauptmann et al. noted that cement thickness was also one of the factors affecting the occurrence of residual bone cement Too much uncured cement is more susceptible to extrusion and deformation during prosthesis implantation. Considering the importance of tibial cement in maintaining the stability of the prosthesis [23–25], too little cement may lead to early loosening of the prosthesis. Therefore, reducing the amount of cement applied on the tibial side might not be the best strategy to prevent residual bone cement. Notably, there is also the possibility of residual bone cement after TKA [26, 27], but the corresponding studies are limited [11, 28, 29]. We hypothesized that different surgical techniques reduce the prevalence of residual bone cement after TKA. Resection of the anterior cruciate ligament (ACL) allows anterior tibial dislocation during TKA. The surgeon was thus able to place the tibial component vertically from above, with the cement spreading uniformly around it (Fig. 2a). Complete extrusion of the cement can ensure a good field of vision for the surgeon. In UKA, the ACL and femoral condyle limit the placement angle of the tibial component, which generates backwards pressure on the cement applied to the tibia and ultimately leads to posterior extrusion of the cement (Fig. 2b).

**Fig. 3:**
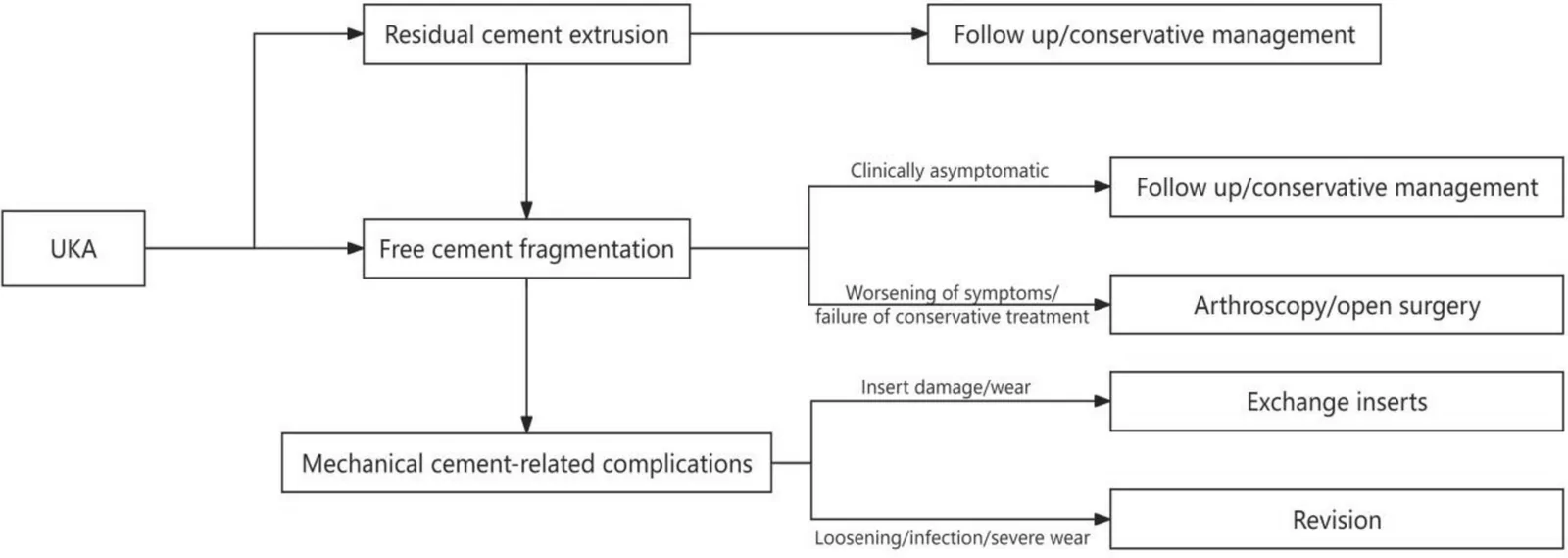
Continuous courses and management strategies for residual bone cement. UKA, unicompartmental knee arthroplasty

The follow-up results of this study revealed that most patients who met the diagnostic criteria for residual bone cement were in the RCE stage. Among patients with FCFs, a portion were clinically asymptomatic; this may be because the FCFs are located in the lateral capsular recess or non-weight-bearing areas and do not participate in articular surface movement. In addition, the small size of the Polyethylene insert is also an important factor (OR 2.59, 95% CI 1.38–4.98, *p* = 0.005). Scheele et al., in a cadaveric knee study, found that regardless of whether hybrid or cemented techniques were used, free cement fragmentation could be detected in 66.7% of UKA specimens [30]. This high prevalence suggests that the presence of small free fragments postoperatively may be a rather “normal” state—most do not cause symptoms. Only a subset of patients with FCFs developed significant pain or limited range of motion. We speculate that, if the knee joint is subjected to frequent and repetitive activity, extruded cement (RCE) could be exposed to sustained mechanical forces. Under such conditions, it might progressively disintegrate and fragment, potentially giving rise to FCF within the joint cavity. The presence of such free bodies could subsequently impede normal flexion and extension, and in severe cases, may even induce joint locking. Nevertheless, this proposed RCE-to-FCF progression remains a hypothesis. Previous studies have noted that particles of bone cement can cause early failure of polyethylene inserts [20], increasing the risk of MCRC. In our six symptomatic patients with FCF, the time from primary surgery to symptom onset ranged from 3 to 48 months. Most of these patients underwent arthroscopic removal of the FCFs. Kim et al. reported a clicking in the knee due to RCE 3 years after UKA [11]. Arthroscopic removal was then performed. The study by Howe et al. included 4 patients who underwent arthroscopic removal due to FCFs [14]. In general, arthroscopic removal is an effective management method with RCE and FCFs. However, Open surgery should be considered when FCFs are located in the posterior articular cavity and cannot be safely retrieved arthroscopically, or when concurrent inspection or exchange of the insert is required. [13]. As a serious complication of UKA, MCRC is relatively rare [6]. Notably, prompt medical treatment is necessary in cases of joint locking or localized pain. A decision chart was designed to synthesize the different states of the residual bone cement and their corresponding management strategies (Fig. 3).

As a preventable phenomenon, residual bone cement has been removed during surgery with a variety of surgical instruments. Keene et al. designed a 90° ball probe to remove extruded cement [31]. There are also reports describing the application of dental mirrors and nerve hooks [6, 14]. In another study, researchers recommended postoperative X-ray detection of residual cement [16]. Changing the implant strategy could also reduce the incidence of residual bone cement. Two packs of cement were used to secure the implants in the femur and tibia, allowing adequate time for the removal of residual cement. The implantation of the tibial prosthesis was advanced, which resulted in fewer visual field obstructions in the posterior region [31], and the femoral trial and a suitable polyethylene spacer were subsequently implanted. The trial-prosthesis combination was passively flexed and extended during surgery. After the removal of the trial and insert, the extruded cement was removed to relieve compression of the tibial prosthesis caused by the implanted shims and the different positions. However, to our knowledge, the current strategy has not been systematically reported. At present, during the procedure, we made three attempts to remove residual bone cement: after implanting the tibial prosthesis, after inserting the twin-peg femoral trial component and the appropriate feeler gauge, and after completing the implantation of all components.

Our study had several limitations. First, it was retrospective and lacked a predefined protocol, which may have introduced selection bias. The small sample size further limited the statistical power of our analyses of residual bone cement. Second, postoperative computed tomography (CT) was performed only in patients with clinical symptoms, whereas asymptomatic patients underwent plain radiography alone; this differential imaging approach likely led to detection bias, potentially underestimating the true prevalence of residual cement. Third, we did not evaluate the Coronal Plane Alignment of the Knee (CPAK) [32] morphotype, which might have confounded the relationship between residual cement and mechanical complications. Fourth, the diagnosis of residual cement was based on imaging data; most affected patients remained asymptomatic, and our cross-sectional design precludes any conclusions regarding the longitudinal progression from residual cement to fibrous fixation or mechanical complications. Finally, despite our intraoperative efforts to minimize this issue, residual cement still occurred more frequently than anticipated. Therefore, extended follow-up is warranted to clarify the natural history of different stages of residual cement and to assess long-term prosthetic survival in this patient population.

## Conclusion

This study retrospectively analyzed the incidence of residual bone cement at various stages following UKA. Routine CT screening was not performed in asymptomatic patients. The true prevalence of residual bone cement following UKA may be higher than currently reported (26.16%), which should not be overlooked. It should be noted that the vast majority of residual bone cement cases were asymptomatic and represented incidental findings on imaging studies. However, once residual cement detaches and forms a FCF, it may provoke mechanical joint symptoms, and a subset of these patients may require surgical intervention. Nevertheless, direct longitudinal evidence is currently lacking regarding whether residual bone cement inevitably leads to clinical symptoms over the course of disease progression, and its long-term risk warrants further investigation. Based on our findings, we propose the following recommendations: intraoperative efforts should focus on enhancing cement removal from the posterior aspect of the tibial component; for patients with thinner polyethylene inserts, more meticulous postoperative imaging screening should be performed to exclude occult residual cement; for FCF presenting with mechanical symptoms, arthroscopic removal may be considered as the initial approach; and insert exchange or revision surgery should be strictly reserved for cases with well-defined indications, including evident polyethylene wear, aseptic loosening, or periprosthetic joint infection.

## Data Availability

The datasets used and/or analysed during the current study are available from the corresponding author on reasonable request.
